# Higher Chain Length Distribution in Debranched Type‐3 Resistant Starches (RS3) Increases TLR Signaling and Supports Dendritic Cell Cytokine Production

**DOI:** 10.1002/mnfr.201801007

**Published:** 2018-11-23

**Authors:** Alexia F.P. Lépine, Roderick H. J. de Hilster, Hans Leemhuis, Lizette Oudhuis, Piet L. Buwalda, Paul de Vos

**Affiliations:** ^1^ Immunoendocrinology Division of Medical Biology Department of Pathology and Medical Biology University of Groningen, University Medical Center Groningen Hanzeplein 1 9700 RB Groningen The Netherlands; ^2^ Food and Biobased Research Wageningen University and Research center Bornse Weilanden 9 6708 WG Wageningen The Netherlands; ^3^ AVEBE Innovation Center P.O.Box 15 9640 AA Veendam The Netherlands; ^4^ Biobased Chemistry and Technology Wageningen University and Research center Bornse Weilanden 9 6708 WG Wageningen The Netherlands

**Keywords:** Caco‐2, dendritic cells, resistant starches, T‐cells, Toll‐like receptors

## Abstract

**Scope:**

Resistant starches (RSs) are classically considered to elicit health benefits through fermentation. However, it is recently shown that RSs can also support health by direct immune interactions. Therefore, it has been hypothesized that the structural traits of RSs might impact the health benefits associated with their consumption.

**Methods and results:**

Effects of crystallinity, molecular weight, and chain length distribution of RSs are determined on immune Toll‐like receptors (TLRs), dendritic cells (DCs), and T‐cell cytokines production. To this end, four type‐3 RSs (RS3) are compared, namely Paselli WFR, JD150, debranched Etenia, and Amylose fraction V, which are extracted from potatoes and enzymatically modified. Dextrose equivalent seems to be the most important feature influencing immune signaling via activation of TLRs. TLR2 and TLR4 are most strongly stimulated. Especially Paselli WFR is a potent activator of multiple receptors. Moreover, the presence of amylose, even to residual levels, enhances DC and T‐cell cytokine responses. Paselli WFR and Amylose fraction V influence T‐cell polarization.

**Conclusions:**

It has been shown here that chain length and particularly dextrose equivalent are critical features for immune activation. This knowledge might lead to tailoring and design of immune‐active RS formulations.

## 1 Introduction

Resistant starches (RSs) are dietary fibers^1^ classically considered to elicit health benefits through fermentation by the gut microbiota^2^ that subsequently enhance production of vitamin B and K but also short chain fatty acids (SCFA) that modulate immunity.^3^ However, it was recently shown that RSs not only serve as substrate for gut‐microbiota but can also support health by directly interacting with specialized receptors on the immune cells.^4^ RSs are polymers of amylose (linear chains) and amylopectin (linear and branched chains) that are an assembly of glucose monomers with alpha‐glucosidic bonds.^5^ RS can be found in different physico‐chemical compositions such as in different crystallinity, particle size, structural order, helicity, molecular weight (MW), and differences in chain‐length distribution, which includes degree of polymerization (DP), dextrose equivalent (DE), and degree of branching. All these factors might influence the interaction of RSs with immune cells or other physiological processes after consumption of RSs. The source, breeding method of the crop, and extraction method determines the physicochemical properties of the RS.^6^, ^7^ There are five types of RS.^5^, ^8^, ^9^ The first type is defined as inaccessible starch, the second as granular starch, the third as retrograded starch, the forth as modified starch, and the fifth as an amylose–lipid complex. Type‐3 (RS3) is obtained by a cooking and cooling process that makes it the most resistant form, fully resistant to human digestive enzymes.^10^ The final physicochemical structural properties of the RS3 are likely to provide direct immune effects.^11^, ^12^

Direct interaction with the immune system is expected to be regulated via binding of RSs to specialized immune cell receptors called pathogen recognition receptors (PRRs) that are expressed on many intestinal cells such as intestinal epithelial cells (IECs) and especially on immune cells in the gut.^13^ As such, RSs have been shown to be able to interact with a subgroup of PRRs called Toll‐like receptors (TLRs).^4^ It has been suggested that particle size might matter but also RS source, crystallinity, DE, and MW might impact the binding to TLR but these have not been studied yet. Although structural features responsible for PRR binding remain to be determined, RSs have been shown to directly modulate cytokine secretion profiles of dendritic cells (DCs) and were able to skew T‐cell polarization.^4^ DCs are known to highly express TLRs and are one of the first immune cell types to come into contact with compounds present in the gut lumen. Their responses were found to be modulated by crosstalk with IECs.^14^, ^15^, ^16^ DCs can subsequently trigger immune responses in other cells, such as T‐cells.^17^ Depending on the inflammatory signaling, T‐cell skewing might be pushed toward regulatory T‐cells (Treg), helper T‐cells (Th) type 1, 2, or 17, each triggering a different immune response.^17^ The cytokines and chemokines secreted upon such activation are specific,^13^ and we hypothesized that they might depend on RS structural traits. Insights in the effector‐structure relationship of RS effects on DCs might lead to tailored RS formulations for specific health benefits.

Here, we determined the effect of crystallinity, MW, and chain length distribution of RS on immune effects of DCs. We first determined the effects of these RS traits on TLR signaling as RS has been reported to bind to these immune receptors.^4^ To this end, we compared four RS3. The short molecular RSs, which were almost exclusively composed of amylopectin, Paselli WFR (WFR), JD150 (JD), and debranched Etenia (dEtenia) were enzymatically debranched^17^; while the large molecular RS Amylose fraction V (AmyloseV) was a fraction of the gelatinized starch that solely contained amylose. First, JD and dEtenia were compared because they predominantly differ in crystallinity; then JD and WFR mostly differed in DE; and finally, AmyloseV strongly differed from the other three RSs at all levels.  However, other parameters also varied aside from these major differences and we therefore compared how combinations of these structural traits might affect immune responses. TLR expressing reporter cells were exposed to all four RSs. Furthermore, effects of RSs on DCs secretory cytokine profile were measured with and without IECs; and the subsequent effect on T‐cell skewing was further evaluated during exposure of immature/naive T‐cells to DCs spent medium (DC‐SM).

## 2 Experimental Section

### 2.1 Characterization of the Resistant Starches

The type‐3 resistant starches (RS3) Paselli WFR (WFR), debranched amylopectin JD150 (JD), debranched Etenia 457 (dEtenia), and Amylose fraction V (AmyloseV; Avebe U.A., Veendam, The Netherlands) were all derived from potato starch. The amylose was separated from potato starch that contained ≈80% amylopectin and ≈20% amylose, as previously described.^18^ Briefly, starch was fractionated by solubilization in a hot magnesium sulphate solution. The amylose fraction precipitated upon cooling whereas the amylopectin remained in the water phase. The other three products, WFR, JD, and dEtenia, were obtained via enzymatic debranching using the pullulanase Promozyme D2 of Novozymes at pH 4.7 and 60 °C. Prior to debranching, the starch was first gelatinized at 160 °C with steam. After cooling down, the pH was set to 4.7 by adding 3 m acetic acid. WFR was prepared from maltodextrin with low DE, JD from starch containing amylopectin only, and dEtenia from Etenia 457. Etenia 457 was made from potato starch, as previously described,^19^ by treating the gelatinized starch with a 4‐α‐glucanotransferase. The four products differed in their crystallinity, chain length distribution (DE and DP), and MW.

The degree of partial crystal structures was determined by enzymatically degrading the noncrystalline fraction. The α‐amylase digestion was performed at 40 °C in 100 mm sodium acetate buffer of pH 5.0 supplemented with 5 mm CaCl_2_ using 6 units mL^–1^ of *Bacillus licheniformis* α‐amylase (E‐BLAAM; Megazyme, Wicklow, Ireland). Subsequently, the supernatant fraction was collected. The partly hydrolyzed starch/amylose present in the supernatant was further hydrolyzed to glucose using amyloglucosidase (Megazyme; E‐AMGDF). The amount of glucose was quantified with the d‐Glucose‐HK kit (Megazyme; K‐GLUHKR). The degree of crystallinity is defined as 100% minus the percentage of glucose formed.

- The DP was assessed by high pH anion exchange chromatography (HPAEC). The products were dissolved in DMSO:milliQ water (9:1) at a concentration of 500 mg L^–1^. The HPAEC‐PAD ICS5000 workstation (ThermoFisher Dionex) was equipped with a Carbo‐Pac PA‐1 column (4 × 250 mm), a PA‐1 guard column and a pulsed amperometric detector with an AgCl/Gold reference electrode. The column was kept at 30 °C. The system was run with a linear gradient of 10 to 400 mm sodium acetate in 100 mm sodium hydroxide at flow rate of 1 mL min^–1^.
- The products differ also in the percentage of reducing ends, which was expressed as DEs where native starch had a DE value of 0 and dextrose of 100. The average molecular weight (Mn) of the reducing sugars in the hydrolyzed fraction of each of the RSs was quantified using the Luff–Schoorl reagent (VWR) and is expressed as DE in g per 100 g. Dextrose, by definition, has a DE of 100 g per 100 g and is the reducing sugar with the lowest MW.

MW analysis was done by size exclusion chromatography (SEC), as previously described,^20^ with minor modifications. Starch samples were dissolved in Dimethylsulfoxide (DMSO)‐LiBr (0.05 m) at a concentration of 2 mg mL^–1^ by overnight rotating at room temperature (RT), followed by 3 h rotation in a ventilation oven at 80 °C. All samples were allowed to cool down to RT, and were then filtered through a 0.45 µm PTFE membrane. The SEC system from PSS (Mainz, Germany) was applied to determine Mn and the polydispersity index, defined as MW/Mn. The system consisted of an isocratic pump, auto sampler without temperature regulation, online degasser, 0.2 µm inline filter, refractive index detector (G1362A 1260 RID Agilent Technologies), viscometer (ETA‐2010 PSS, Mainz), and MALLS detector (SLD 7000, PSS, Mainz). DMSO‐LiBr (0.05 M) was employed as the eluent. The samples were injected at a flow rate of 0.5 mL min^–1^ into a MZ Super‐FG 100 SEC column and two PFG SEC columns 300 and 4000. The columns were kept at 80 °C, the viscometer at 60 °C, and the refractive indicator at 45 °C. A universal calibration curve was obtained using the pullulan standard kit (PSS, Mainz, Germany) with MW ranging from 342 to 805 000 Da. Data were processed with the WinGPC Unity software (PSS, Mainz). A refractive index increment (dn/dc) value of 0.072 was used. DMSO (CHROMASOLV Plus, HPLC grade, > 99.7%) was purchased from Sigma Chemical Company and anhydrous lithium bromide (99%) from Fisher Scientific. Note that the MW of AmyloseV was obtained via the MALLLS detector, which was not possible for the other compounds as they do not cause enough light scattering. Instead, the MW of JD, WFR, and dEtenia was estimated from the calibration curve obtained with the pullulan standard kit.

Contamination in DNA and RNA of the starch products was evaluated by using NanoDrop 1000 Spectrophotometer (Thermo Scientific, Breda, The Netherlands). This confirmed low levels of such contaminants. DNA was present in WFR (13.71 ng µL^–1^) and AmyloseV (65.89 ng µL^–1^), and RNA was found in AmyloseV (41.89 ng µL^–1^) and dEtenia (6.33 ng µL^–1^). Furthermore, contamination by endotoxins was measured using the Limulus Amoebocyte Lysate (LAL) to quantitatively determine the amount of LPS present in the samples. The LAL assay was carried out according to the manufacturers protocol from Pierce LAL Chromogenic Endotoxin Quantitation Kit, Thermo Scientific (Pierce Biotechnology, Rockford, USA). In short, the microplate was brought to 37 °C and 50 µL of standard or sample were added. After 5 min incubation, 50 µL of LAL reagent was added followed by another 10 min incubation. Then 100 µL of substrate solution was added and the microplate was further incubated for 6 min, after which the reaction was stopped with 25% acetic acid. Absorbance was measured at 405–410 nm on a Bio‐Rad Benchmark Plus microplate spectrophotometer reader (Bio‐Rad Laboratories B.V, Veenendaal, the Netherlands) using Bio‐Rad Microplate Manager 5.2.1 software. The average absorbance of each samples was then calculated based on the standard curve and concentration was expressed in ELISA Unit (EU) mL^–1^, which was approximately 0.1 ng endotoxin mL^–1^. LPS was not detected in JD and very low amounts were present in dEtenia (0.076 EU mL^–1^). On the other hand, WFR and AmyloseV contained higher amounts of LPS, respectively, 0.961 and 0.728 EU mL^–1^.

### 2.2 Cell Culturing

THP‐1‐blue monocyte reporter and Human Embryonic Kidney (HEK)‐blue cell lines were cultured and used as previously described.^21^ THP‐1 were cultured in RPMI1640 medium (Gibco, Life Technologies, Bleiswijk, The Netherlands), containing 10% heat inactivated Fetal calf Serum (hiFCS) (HyClone, Thermo Scientific, Breda, The Netherlands), 2 mm l‐glutamine, 4.5 g L^–1^ glucose, 100 mg mL^–1^ Normocin (InvivoGen, Toulouse, France), 10 mm HEPES, 1.0 mm sodium pyruvate, 1.5 g L^–1^ sodium bicarbonate (Boom B.V. Meppel, The Netherlands), and 0.5% penicillin–streptomycin (50 µg mL^–1^–50 µg mL^–1^). THP‐1 cell lines were passaged twice a week.

HEK‐blue cells were cultured using DMEM containing 10% hiFCS, 2 mm l‐glutamine, 4.5 g L^–1^ glucose, 0.2% Normocin, and 0.5% penicillin–streptomycin (50 µg mL^–1^–50 µg mL^–1^). The cells were passaged when confluency reached 50–90%.

ATCC derived Caco‐2 (HTB‐37, 2012) cells were cultured as previously described^22^ in Dulbecco's modified Eagle's medium (DMEM; Gibco‐Invitrogen, Bleiswijk, The Netherlands) supplemented with 10% hiFCS (HyClone), 4.5 g L^–1^ glucose, 0.58 g L^–1^ glutamine, 10% MEM nonessential amino acids (Gibco, Thermo Fischer Scientific, Waltham, MA, USA), 0.5% penicillin–streptomycin (50 µg mL^–1^–50 µg mL^–1^) and no pyruvate. Cells were used within passage numbers 25 to 40.

Primary DCs and autologous T‐cells were purchased from MatTek Corporation (Ashland, MA, USA) and cultured according to manufacturer's instructions. Briefly, cells were thawed in specific basal medium (MatTek), centrifuged at 250 × *g* for 10 min, pellet was resuspended in specific maintenance medium (MatTek), and seeded at the appropriate concentration as described below.

### 2.3 Reporter Cells Activation and Inhibition Assays

THP‐1‐blue cell line was used as previously described^21^ to determine possible interaction of RSs with several PRRs. THP‐1 monocytes express some PRR subtypes such as Toll‐like receptor (TLR) and NOD‐like receptors (NLR). THP‐1‐blue cells are derived from the human THP‐1 monocyte cell line and carry an NF‐κB‐inducible Secreted Embryonic Alkaline Phosphatase (SEAP) reporter construct allowing the monitoring of NF‐κB activation by determining the activity of SEAP. This cell line also carries an extra insert for MD2 and CD14 that boosts TLR signaling. Levels of NF‐κB‐induced SEAP in the cell culture supernatant were measured with the detection reagent QUANTI‐Blue (InvivoGen, Toulouse, France). The essential downstream signaling protein of most TLR subtypes is MyD88. Inhibition of this protein therefore leads to blockage of the TLR‐dependent NF‐κB pathway. The inhibition was induced using 50 µm Pepinh‐MyD88 (InvivoGen).

THP‐1 activation and inhibition protocols were performed according to manufacturers’ instructions. Cell were seeded at a density of 1 × 10^6^ per well and the agonist LPS (0.1 µg mL^–1^) was used as a positive control. The THP‐1 cells were incubated with the pepinh‐MyD88 for 6 h prior to exposure to the compounds. After 24 h stimulation at 37 °C 5% CO_2_, the secreted SEAP was measured by adding QUANTI‐Blue and absorbance was measured at 650 nm after 1 h with the Bio‐Rad Benchmark Plus microplate spectrophotometer reader (Bio‐Rad Laboratories B.V). Each condition was performed in triplicate and experiments were performed three times on different days.

Specific interaction of RSs with TLRs was investigated, as previously described,^21^ using HEK‐blue cells overexpressing either TLR2, 3, 4, 5, 7, 8, or 9. HEK‐blue, as THP‐1‐blue cells, carried an NF‐κB‐inducible SEAP reporter construct that allowed for quantification of TLR activation using QUANTI‐Blue. The HEK‐blue cells were seeded according to manufacturer's protocol as described in **Table** 1. The cells were kept O/N at 37 °C 5% CO_2_ to attach to the plate, after which medium was replaced with medium containing 5, 1, or 0.5 mg mL^–1^ of each RS. Culture medium was used as a negative control and specific agonists were used as positive controls according to Table 1. In the case of TLR4 activation, 100 µg mL^–1^ polymyxin B was added to capture any LPS present in the samples as LAL assay revealed contamination in two of the four RSs.^23^ TLR inhibition assays consisted of adding 10 µL of TLR specific agonist together with the RSs to investigate possible interactions between the two. Activation experiments included six replicates, inhibition experiments included triplicates, and each of these experiments was repeated at least five times, on different days.

**Table 1:** Cell seeding densities, positive controls, and final concentrations used in reporter cell stimulations

HEK‐Blue cell line overexpressing	Selection antibiotics	Cell density [cells mL^–1^]	Positive control (agonist)
TLR2	HEK‐Blue (1 µL mL^–1^)	2.8 × 10^5^	Heat‐killed Listeria Monocytogenes (HKLM, 10^8^ cells mL^–1^)
TLR3	Blasticidin (30 µg mL^–1^) Zeocin (100 µg mL^–1^)	2.8 × 10^5^	Poly (I:C) Low Molecular Weight (LMW, 1 µg mL^–1^)
TLR4	HEK‐Blue (1 µL mL^–1^)	1.4 × 10^5^	*E. coli* K12 Lipopolysaccharide‐HEK Ultrapure (LPS, 0.1 µg mL^–1^)
TLR5	Blasticidin (30 µg mL^–1^) Zeocin (100 µg mL^–1^)	1.4 × 10^5^	Recombinant flagellin isolated from *S*. Typhimurium (RecFLA‐ST, 0.1 µg mL^–1^)
TLR7	Blasticidin (10 µg mL^–1^) Zeocin (100 µg mL^–1^)	2.2 × 10^5^	9‐Benzyl‐8 hydroxyadenine derivative (CL264, 5 µg mL^–1^)
TLR8	Blasticidin (30 µg mL^–1^) Zeocin (100 µg mL^–1^)	2.2 × 10^5^	20‐mer Phosphorothioate single stranded RNA is complexed with the transfection reagent LyoVec (ssRNA40/LyoVec, 2 µg mL^–1^)
TLR9	Blasticidin (10 µg mL^–1^) Zeocin (100 µg mL^–1^)	4.5 × 10^5^	Type B CpG oligonucleotide (ODN2006, 10 µM µM)

### 2.4 DC and T‐Cells Stimulation

**Figure 1:**
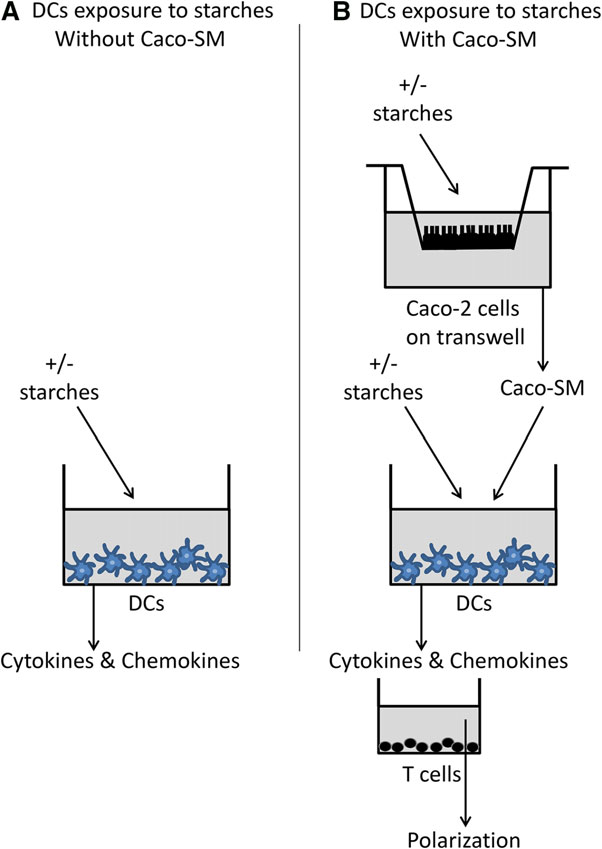
Experimental design for DCs stimulation with the RSs JD, WFR, dEtenia, and AmyloseV, in absence (A) and presence (B) of Caco‐SM. Effects of RS3 on dendritic cells (DCs) directly exposed to these ingredients for 20 h (A) were compared in a model for intestinal cellular cross‐talk, where DCs were also exposed to the spent medium collected from Caco‐2 cells (Caco‐SM).^21^ For this purpose, Caco‐2 cells were cultured on transwells for 21 days in a separate plate and were incubated for 20 h with the RSs. The Caco‐SM collected was then transferred to the DCs that were separately cultured (B). In this setting, DCs were exposed concomitantly to this Caco‐SM and to the corresponding, freshly prepared, RS. At last, T‐cells cultured in a separate plate were exposed for 24 h, in a 1:10 ratio, to the DC‐SM.

To evaluate the direct immune effects of RSs on intestinal cells, an IEC‐DC coculture system was used that was compared to separate IECs and DCs stimulations (**Figure** 1). These experiments were performed as previously described^21^ and all experiments were performed five times, on different days. For these experiments, Caco‐2 cells were seeded at a density of 330 000 cells per well of a ThinCert transwell with 33.6 mm^2^ membranes and 3 µm pore size (Geiner Bio‐One International GmbH, Alphen aan den rijn, the Netherlands), and differentiated for 21 days at 37 °C 5% CO_2_. Medium was refreshed three times a week and on the day prior to stimulation with the RSs. Integrity of the Caco‐2 monolayer was confirmed by measuring trans‐epithelial electric resistance (TEER) prior and post exposure to RSs.

DCs seeded onto a 96‐well plate (40 000 DCs per well) were left to adhere O/N and were then exposed to 5mg mL^–1^ of RSs for 20 h. Culture supernatants of triplicates were pooled and stored at –80 °C until further exposure to T‐cells or analysis. Next, a similar experiment was carried out in the same way except that DCs were exposed to the freshly prepared RSs combined to Caco‐SM collected on beforehand. The apical surface of the Caco‐2 IEC monolayers was stimulated with Caco‐2 medium or 5 mg mL^–1^ RSs. The plates were incubated 24 h at 37 °C 5% CO_2_ and basolateral supernatants of the triplicates were pooled and used for further exposure to DCs. After 20 h stimulation, DC‐SM of triplicates were pooled and stored at –80 °C until further exposure to T‐cells or analysis.

T‐cells autologous to the DCs were seeded onto a 96‐well plate (40 000 T‐cells per well), let to adhere O/N and exposed to DC‐SM, diluted 1:10, for 24 h at 37 °C 5% CO_2_. Culture supernatants of triplicates were pooled and stored at –80 °C until further analysis.

### 2.5 Cytokine Expression Analyses

The cytokines and chemokines produced by DCs gave an indication about possible T‐cell skewing as response to exposure to RSs. To measure the levels of this signaling molecules in the spent medium collected from DCs (DC‐SM), exposed or not Caco‐SM, and T‐cells, a Magnetic Luminex premixed cytokine assay (R&D systems Inc, Minneapolis, USA) was used. This kit was customized to simultaneously measure the following molecules in CC‐SM and DC‐SM: IL‐12/23 p40, IL‐1Ra, IL‐1β, IL‐6, MCP‐1/CCL2, CCL3/MIP‐1α, CCL‐5/RANTES, IFN‐γ, IL‐10, and TNF‐α; and the following molecules in T‐cell supernatant: IL‐2, IL‐4, IL‐6, IL‐10, IL‐17A, IFN‐γ, and TNF‐α. Besides, IL‐8 in 1:4 diluted DC‐SM was measured separately using a human CXCL8/IL‐8 DuoSet ELISA kit (R&D systems).

Luminex assays were performed according to the manufacturer's instructions. Briefly, a concentration series of cytokine standards were prepared for the appropriate concentration range. The undiluted microparticle reagent mix specific for either DCs or T‐cells was added to each well (50 µL per well), washed, and standards, negative controls, and samples were all incubated O/N at 4 °C shaking (duplos, 50 µL per well). After incubation, the plate was washed three times, a biotin antibody cocktail was added to each well (50 µL per well) and the plate was further incubated while shaking for 1 h at RT. The plate was washed three times and streptavidin–phycoerythrin was added to each well (50 µL per well). After 30 min incubation shaking at RT, the plate was washed three times and the microparticles were resuspended in 100 µL of wash buffer. Fluorescence was then measured within 90 min using a Luminex analyzer MAGPIX and xPONENT 4.2 for MAGPIX software (Luminex Corporation, ‘s‐Hertogenbosch, The Netherlands).

### 2.6 Statistical Analysis

THP‐1 and cytokine data were expressed as average with SEM and HEK‐blue data were expressed as average with SD. Statistical analyses were performed using GraphPad Prism version 7.0a (GraphPad Software, Inc., La Jolla, USA). Normality was assessed using D'Agostino & Pearson and ANOVA test was then used and followed by LSD for THP‐1 and cytokines profiles, or Kruskal–Wallis test was used followed by Dunn's multiple comparison for HEK‐blue results. Differences between RSs and medium or agonist controls were considered statistically significant when *p < *0.05, and *p *< 0.1 is a trend. *p*‐Values < 0.05 are denoted with *, *p *< 0.01 with **, *p *< 0.001 with ***, and *p *< 0.0001 with ****.

## 3 Results

### 3.1 Characterization of Type‐3 Resistant Starches

The structural properties of the four small molecular RS3, namely WFR, JD, dEtenia, and of the larger RS AmyloseV are summarized in **Table** 2.

**Table 2:** Summary of compound chemical and structural analysis. Crystallinity levels, chain length distribution, as represented by dextrose equivalent and degree of polymerization,and molecular weight are detailed for each of the four RS3 analyzed

	Crystallinity (%a))	Chain length distribution		Molecular weight (10^3^ g mol^–1^ b))
		Dextrose equivalent (g per 100 g)	Degree of polymerization	
Paselli WFR	7	8.1	1–27	2
JD150	7	4.2	6–20	2
Debranched Etenia	14	4.1	1–22	4
Amylose fraction V	92	1.2	NA	200

^a)^Crystallinity refers here to the percentage of starch that resists enzymatic digestion as crystalline material is expected to be formed upon cooling of RS3.^24^

^b)^The MW of AmyloseV is obtained from light scattering, and the MW of WFR, JD150, and dEtenia are estimated by comparison to a pullulan standard.

Gelatinized starches start to retrograde upon cooling, resulting in partly crystalline material^24^ thus containing crystalline material resisting enzymatic hydrolysis.^1^ Crystallinity of the RSs was determined by quantifying the time required by α‐amylase to degrade the RSs at 40 °C (Table 2). WFR and JD both contained 7% of crystalline structures, dEtenia contained 14%, and AmyloseV contained 92%.

The DE was also quantified as this value is a measure of the amount of starch molecules, because each starch molecule carries one reducing end. As such, the DE gives the Mn of the products obtained after hydrolysis of the RSs.^25^ It showed that WFR had the highest DE (8.1 g per 100 g), whereas JD and dEtenia were intermediate (respectively, 4.2 g per 100 g and 4.4 g per 100 g), and AmyloseV had the lowest DE (1.2 g per 100 g) (Table 2).

The chain length distribution analysis of the short products, i.e., WFR, JD, and dEtenia, revealed that WFR and dEtenia had a more uniform distribution. The range of WFR was DP1‐27 with the highest abundance within DP6‐16. dEtenia had a uniform distribution within DP1‐22, whereas JD had a peak at oligosaccharides in the DP range 10–18 with the highest abundance within DP6‐16, and also contained glucose (DP1; Table 2).

Finally, the MW of the starches were compared using GPC‐MALLS‐RI in DMSO. AmyloseV had by far the highest MW, estimated to be ≈200 000 g mol^–1^, whereas WFR, JD, and dEtenia had much lower MW in the range of 2000–4000 g mol^–1^ (Table 2).

### 3.2 JD and dEtenia Stimulate TLR4 But Stimulation is Not Dependent on Crystallinity

The starches JD and dEtenia originate from the exact same native starch source and underwent enzymatic debranching. The only difference during the production of these two compounds was the cooling process. Therefore, the crystallinity of JD was around 7% and that of dEtenia was double while other structural traits were similar (Table 2). As these two RSs differ predominantly in crystallinity, they were used for determining possible effects of this characteristic on immune signaling.

**Figure 2:**
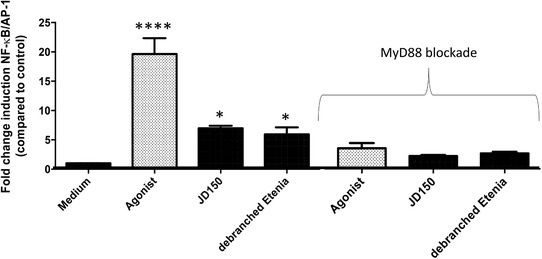
RSs JD150 and debranched Etenia stimulated monocytic THP‐1 cells in a TLR dependent manner. To determine whether immune activation of the two RSs JD and dEtenia was TLR dependent, we determined activation of THP‐1 cells in fully functional THP‐1 cells (left). We also used THP‐1 cells where MyD88 was inhibited by 50 µm Pepinh‐MyD88 (right, as indicated by ‘MyD88 blockade’). Data were normalized so that medium control is 1, and activation levels were expressed as fold change induction of NF‐κB/AP‐1 pathway as compared to medium control ± SEM with *n* = 3 and triplicates. Data were analyzed using GraphPad Prism ANOVA test followed by LSD test, and differences were considered statistically significant when *p < 0.05, **p < 0.01, ***p < 0.001, and ****p < 0.0001.

As RSs might influence TLR signaling,^4^ we first compared effects of the two RSs with different crystallinity on TLR activation. First, we determined whether these two RSs signal via TLRs. This was done by incubating JD and dEtenia at a concentration of 5 mg mL^–1^ with THP‐1 monocytes, a cell line that expresses all TLR subtypes (**Figure** 2). To evaluate if activation of THP‐1 is solely TLR‐mediated, we also performed this experiment during blockade of the essential downstream signaling protein MyD88.

**Figure 3:**
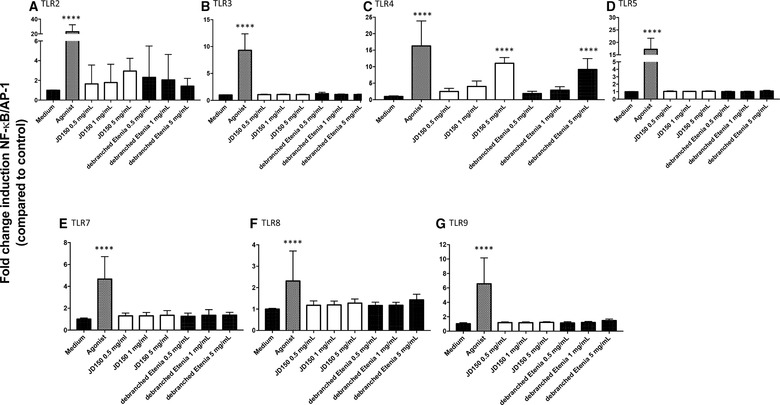
Effect of crystallinity on TLR signaling: RSs JD150 and debranched Etenia only activated TLR4, which was not influenced by their crystallinity degree. RS JD150 had a crystallinity of 7% and debranched Etenia (dEtenia) of 14%. TLR signaling was determined by adding these two RSs to various HEK‐cells expressing only one TLR: TLR2 (A), TLR3 (B), TLR4 (C), TLR5 (D), TLR7 (E), TLR8 (F), and TLR9 (G). Both JD and dEtenia were shown to stimulate TLR4 (C). The agonist used was LPS. The activation levels triggered by the RSs were compared to the medium control. Data were normalized so that medium control is 1, and activation levels were expressed as fold change induction of NF‐κB/AP‐1 pathway as compared to medium control ± SD with *n* = 5 and triplicates. Data were analyzed with GraphPad Prism Kruskal–Wallis test followed by Dunn's multiple comparison and differences were considered statistically significant when *p<0.05, **p < 0.01, *p *< 0.001, and *p *< 0.0001.

Both JD (sevenfold; *p =* 0.017) and dEtenia (sixfold; *p =* 0.046) significantly activated THP‐1 cells (Figure 2A). In presence of the MyD88 blocker Pepinh‐MyD88, this activation was lost for both RSs (Figure 2B), confirming TLR dependency. Next, to determine which TLR was activated, we tested the two RSs on HEK‐Blue reporter cell lines that only express one TLR. We also assessed possible dose‐dependent effects by testing three different concentrations of each RS: 0.5, 1, and 5 mg mL^–1^. Only TLR4 was activated by these two RSs at 5 mg mL^–1^ (**Figure** 3C). However, no difference could be observed between JD and dEtenia. No inhibition could be observed for either of these two RSs.

To exclude that the TLR4 activation of the two starches was caused by a possible contamination with LPS, we added 100 µg mL^–1^ polymyxin B to the RSs to capture any LPS present in the samples.^23^ As shown in Figure S1, Supporting Information, addition of polymyxin B fully blocked the effect of LPS agonist control (Figure S1A, Supporting Information) but only had a small reducing effect on TLR4 activation by JD (Figure S1B, Supporting Information) and dEtenia (Figure S1C, Supporting Information). The activation was still significant. This demonstrates that the observed TLR4 effects were truly induced by the RSs.

Next, to determine whether the TLR activation has any functional meaning, we tested whether the two RSs JD and dEtenia can trigger different responses in dendritic cells (DCs), which highly express TLRs.^26^ DCs encounter starches in the intestine via extruding their dendrites through the epithelial layer.^27^ The secretion of the human cytokines and chemokines IL‐12/23 p40, IL‐1Ra, IL‐1β, IL‐6, IL‐8, MCP‐1/CCL2, CCL3/MIP‐1α, CCL‐5/RANTES, IFN‐γ, IL‐10, and TNF‐α was measured after 20 h exposure of DCs to 5 mg mL^–1^ of the two RSs. The production of IL‐1β, CCL3/MIP‐1α, CCL‐5/RANTES, IFN‐γ, IL‐12.23p40, and IL‐10 was below detection levels at all occasions. As shown in **Table** 3, dEtenia had no effect on DCs while JD increased IL‐8 production (*p* = 0.007).

**Table 3:** Lower crystallinity of JD increases IL‐8 production by DCs. The effects of JD150 and debranched Etenia on IL‐1Ra, IL‐6, IL‐8, MCP‐1/CCL2, and TNF‐α production was measure by Luminex in spent medium of DCs exposed to RSs directly, DCs exposed to RSs and to Caco‐SM after Caco‐2 were themselves exposed to the RSs, DCs exposed to Caco‐2 spent medium after Caco‐2 were exposed to the RSs and spent medium from the basolateral compartment of the coculture of DCs with Caco‐2 cells

	Average (pg mL^–1^)	SEM	Average (pg mL^–1^)	SEM	Average (pg mL^–1^)	SEM
**DC‐SM Ingredients**	Medium		JD150		Debranched Etenia	
CCL‐2/MCP‐1	113.2	7.8	177.1	14.7	128.0	11.2
IL‐1ra	1852.0	126.4	2987.0	392.9	2677.0	335.1
IL‐6	5.8	2.1	18.9	6.8	7.6	1.8
IL‐12/23p40	29.0	0.0	68.1	7.7	52.8	0.0
TNF‐a	6.9	1.0	41.3	6.9	51.9	10.8
IL‐8	420.3	18.0	**1401.0** **	160.0	949.6	75.3
**DC‐SM Caco‐SM with ingredients**	Medium		JD150		Debranched Etenia	
CCL‐2/MCP‐1	116.8	5.1	168.7	18.5	131.7	12.7
IL‐1ra	1733.0	153.3	**3043.0** *	292.3	**3367** ***	363.0
IL‐6	2.9	0.2	9.7	2.9	5.1	0.9
IL‐12/23p40			**73.4** ***	6.0	**57.5** ***	5.5
TNF‐a	8.7	0.7	49.9	8.2	**65.7** #	7.4
IL‐8	498.4	25.7	**1199.0** *	108.2	917.0	78.4

Caco‐2 cells fully differentiated for 21 days in transwells were stimulated apically with the different compounds at 5 mg mL^–1^ for 20 h. In direct exposure of RSs to the DCs, 5 mg mL^–1^ was used and incubation time was 20 h. The data shown are the average with SEM values of five repetitions, each including triplicates. Statistical significances were tested in GraphPad Prism ANOVA followed by LSD and ^#^indicates a trend when *p* < 0.1, significances are indicated with **** when *p *< 0.0001, **p < 0.01, and *p < 0.05 when compared to control DCs unstimulated in the case of direct stimulation of DCs by RSs, or compared to control DCs exposed to unstimulated Caco‐SM.

As it is recognized that the crosstalk between DCs and IECs is essential for maintaining gut homeostasis and DC phenotype, we investigated how IECs, stimulated with starches, can impact DC behavior across an epithelial barrier.^4^, ^14^, ^28^ Therefore, we repeated this experiment and exposed DCs to one of the RSs together with the Caco‐2 spent medium (Caco‐SM) collected after Caco‐2 cells were themselves exposed to that same RS for 20 h (Figure 1). Statistically significant differences were observed in production of cytokines and chemokines by DCs exposed to this combination for 20 h (Table 3). The production of IL‐1β, CCL3/MIP‐1α, CCL‐5/RANTES, IFN‐γ, IL‐12.23p40, and IL‐10 was below detection levels at all occasions. JD increased IL‐1ra (*p* = 0.003), IL‐12/23p40 (*p *< 0.0001), and IL‐8 (*p* = 0.003). dEtenia increased IL‐1ra (*p* = 0.0003), IL‐12/23p40 (*p *< 0.0001), and TNF‐α (*p* = 0.025). No statistically significant difference was observed between the two RSs.

Finally, we evaluated the impact of DC responses to the RSs on T‐cell polarization. To this end, T‐cells were exposed to 1:10 ratio of DC‐SM. The human Th1 cytokines IL‐2, IFN‐γ, and TNF‐α; Th2 cytokines IL‐4 and IL‐6; Th17 cytokine IL‐17; and Treg cytokine IL‐10 were measured after T‐cells were exposed to DC‐SM for 20 h (**Table** 4). The production of IL‐17, IFN‐ γ, and TNF‐α was below detection levels at all occasion. No statistically significant difference could be observed.

**Table 4:** Crystallinity does not change T‐cells polarization as JD and dEtenia have virtually no effect on T‐cells at all times. The effects of JD150 and debranched Etenia on IL‐2, IL‐4, IL‐6, and IL‐10 production was measure by Luminex in spent medium of T‐cells exposed to DCs or co‐culture spent medium

	Average (pg mL^–1^)	SEM	Average (pg mL^–1^)	SEM	Average (pg mL^–1^)	SEM
**T‐cell DC‐SM Caco‐SM with ingredients**	Medium		JD150		Debranched Etenia	
IL‐2	66.0	2.9	70.0	3.5	65.8	2.2
IL‐4	2507.0	197.4	2499.0	165.9	2512.0	185.0
IL‐6	1.7	0.5	4.3	0.8	0.9	0.1
IL‐10	29.4	5.0	30.6	3.9	35.3	5.2

T‐cells were incubated with spent medium in a 1:10 ratio for 20 h. The data shown are the average with SEM values of five repetitions, each including triplicates. Statistical significances were tested in GraphPad Prism ANOVA followed by LSD and compared to control T‐cells.

Comparing the RSs JD and dEtenia that have different crystallinity levels showed that this factor did not influence the magnitude of activation of TLRs.

### 3.3 Higher Dextrose Equivalent is Associated with Stronger Immune Activating Effects

Next, we compared the two RSs WFR and JD that both have a crystallinity of 7% but greatly differ in chain length distribution, as DE of WFR is 8 g per 100 g, which is double compared to JD (Table 2). We therefore applied the same screening platform to identify the possible influence of combined higher DE and DP on immune activities of these two RSs.

**Figure 4:**
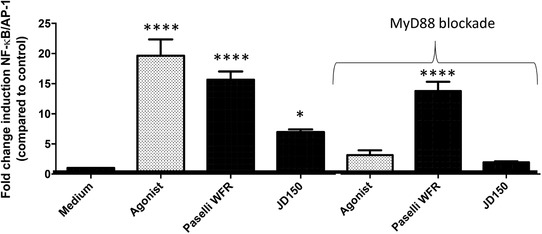
RSs WFR and JD150 differentially stimulated monocytic THP‐1 cells. To determine whether immune activation of the two RSs WFR and JD was TLR dependent, we determined activation of THP‐1 cells in fully functional THP‐1 cells (left). We also used THP‐1 cells where MyD88 was inhibited by 50 µm Pepinh‐MyD88 (right, as indicated by ‘MyD88 blockade’). Data were normalized so that medium control is 1, and activation levels were expressed as fold change induction of NF‐κB/AP‐1 pathway as compared to medium control ± SEM with *n* = 3 and triplicates. Data were analyzed using GraphPad Prism ANOVA test followed by LSD test, and differences were considered statistically significant when *p < 0.05, **p < 0.01, ***p < 0.001, and ****p < 0.0001.

**Figure 5:**
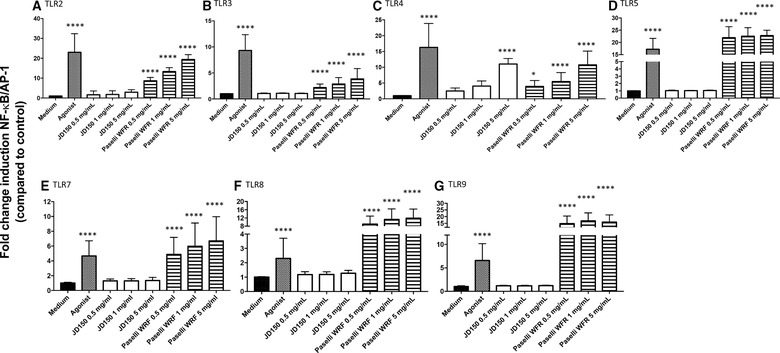
Effect of DE on TLR activation: RS Paselli WFR virtually activated all TLRs. WFR had a DE of 8 g per 100 g and JD150 of 4 g per 100 g. TLR signaling was determined by adding these two RSs to various HEK‐cells expressing only one TLR: TLR2 (A), TLR3 (B), TLR4 (C), TLR5 (D), TLR7 (E), TLR8 (F), and TLR9 (G). WFR activated all TLRs and JD was TLR4 (C) dependent only. The activation levels triggered by the RSs were compared to the medium control. Data were normalized so that medium control is 1, and activation levels were expressed as fold change induction of NF‐κB/AP‐1 pathway as compared to medium control ± SD with *n* = 5 and triplicates. Data were analyzed with GraphPad Prism Kruskal–Wallis test followed by Dunn's multiple comparison and differences were considered statistically significant when *p < 0.05, **p < 0.01, ***p < 0.001, and ****p < 0.0001.

Activation of THP‐1 by WFR reached a 16‐fold (*p *< 0.0001) increase, double of JD (**Figure** 4A). Unlike JD, WFR still activated THP‐1 cells when these were blocked with Pepinh‐MyD88 (Figure 4B). While JD activated TLR4 only (**Figure** 5C), and only at 5 mg mL^–1^, WFR activated all tested TLRs (Figure 5A–G) in a dose‐dependent manner. Activation by WFR was at least as high as the agonist control, except for TLR3 (Figure 5B) and TLR4 (Figure 5C).

To exclude that the TLR4 activation of the two starches was caused by a possible contamination with LPS, we added 100 µg mL^–1^ polymyxin B to the RSs to capture any LPS present in the samples (Figure S1A, Supporting Information), and although addition of polymyxin B had a small impact of TLR4 activation by JD (Figure S1B, Supporting Information) and WFR (Figure S2, Supporting Information), the remaining activation was still statistically significantly higher than medium control. This demonstrates that the observed TLR4 effects were predominantly caused by the RSs themselves.

Also, we determined effects of the differences in starch structure, in this case DE difference, on DC responses. The secretion of the human cytokines and chemokines IL‐12/23 p40, IL‐1Ra, IL‐1β, IL‐6, IL‐8, MCP‐1/CCL2, CCL3/MIP‐1α, CCL‐5/RANTES, IFN‐γ, IL‐10, and TNF‐α was measured after 20 h exposure of DCs to 5 mg mL^–1^ of the two RSs. The production of IL‐1β, CCL3/MIP‐1α, CCL‐5/RANTES, IFN‐γ, IL‐12.23p40, and IL‐10 was below detection levels at all occasions. As shown in **Table** 5, average production of chemokine CCL‐2/MCP‐1 (*p* = 0.004) and pro‐inflammatory IL‐8 (*p *< 0.0001) were significantly higher in DCs exposed to WFR as compared to JD. Besides, WFR, and not JD, increased the production of the pleiotropic cytokine IL‐6 (*p* = 0.031) by DCs.

**Table 5:** Higher DE of WFR greatly increased response of DCs. The effects of Paselli WFR and JD150 on IL‐1Ra, IL‐6, IL‐8, MCP‐1/CCL2, and TNF‐α production was measure by Luminex in spent medium of DCs exposed to RSs directly, DCs exposed to RSs and to Caco‐SM after Caco‐2 were themselves exposed to the RSs, DCs exposed to Caco‐SM after Caco‐2 were exposed to the RSs, and spent medium from the basolateral compartment of the coculture of DCs with Caco‐2 cells

	Average (pg mL^–1^)	SEM	Average (pg mL^–1^)	SEM	Average (pg mL^–1^)	SEM
**DC‐SM Ingredients**	Medium		Paselli WFR		JD150	
CCL‐2/MCP‐1	113.2	7.8	**344.6** ***	44.8	177.1	14.7
IL‐1ra	1852.0	126.4	2661.0	239.1	2987.0	392.9
IL‐6	5.8	2.1	**73.2** *	24.6	18.9	6.8
IL‐12/23p40	29.0	0.0	132.0	25.2	68.1	7.7
TNF‐a	6.9	1.0	98.1	15.9	41.3	6.9
IL‐8	420.3	18.0	**3235.0** ****	388.5	**1401.0** **	160.0
**DC‐SM Caco‐SM with ingredients**	Medium		Paselli WFR		JD150	
CCL‐2/MCP‐1	116.8	5.1	**357.0** ****	56.4	168.7	18.5
IL‐1ra	1733.0	153.3	**3276.0** ****	233.3	**3043.0** *	292.3
IL‐6	2.9	0.2	**36.6** ****	6.5	9.7	2.9
IL‐12/23p40			**187.1** ****	10.8	**73.4** ****	6.0
TNF‐a	8.7	0.7	**126.8** ****	22.1	49.9	8.2
IL‐8	498.4	25.7	**3163.0** ****	381.7	**1199.0** *	108.2

Caco‐2 cells fully differentiated for 21 days in transwells were stimulated apically with the different compounds at 5 mg mL^–1^ for 20 h. In direct exposure of RSs to the DCs, 5 mg mL^–1^ was used and incubation was 20 h. The data shown are the average with SD values of five repetitions, each including triplicates. Statistical significances were tested in GraphPad Prsim ANOVA followed by LSD and ****indicates *p *< 0.0001, ***p < 0.001, **p < 0.01, and *p < 0.05 when compared to unstimulated DCs.

The effect of WFR was more pronounced even when DCs were exposed to both the RSs and Caco‐SM (Table 5), mimicking the situation in the gut where the RS first encounters IECs. The production of IL‐1β, CCL3/MIP‐1α, CCL‐5/RANTES, IFN‐γ, and IL‐10 was below detection levels at all occasions. Many cytokines differed between WFR and JD (Table 5). The average increased production of IL‐1ra (*p* = 0.0002), CCL‐2 (*p*<0.0001), and IL‐6 (*p* = 0.0006) was higher when DCs were exposed to WFR as compared to JD. WFR, but not JD, increased production of TNF‐α (*p *< 0.0001).

Finally, there was some effect of WFR on T‐cell polarization as presented in **Table** 6. WFR increased IL‐6 (*p* = 0.001) production by T‐cells when exposed to the DC‐SM. This average increased production of IL‐6 was statistically significantly higher in DCs exposed to WFR as compared to JD (*p* = 0.016).

**Table 6:** Higher DE of WFR increased IL‐6 production by T‐cells. The effects of Paselli WFR and JD150 on IL‐2, IL‐4, IL‐6, and IL‐10 production were measured by Luminex in spent medium of T‐cells exposed to DCs or coculture spent medium

	Average (pg mL^–1^)	SEM	Average (pg mL^–1^)	SEM	Average (pg mL^–1^)	SEM
**T‐cell DC‐SM Caco‐SM with ingredients**	Medium		Paselli WFR		JD150	
IL‐2	66.0	2.9	65.1	2.3	70.0	3.5
IL‐4	2507.0	197.4	2606.0	186.5	2499.0	165.9
IL‐6	1.7	0.5	**9.1** **	2.5	4.3	0.8
IL‐10	29.4	5.0	34.0	5.3	30.6	3.9

T‐cells were incubated with spent medium in a 1:10 ratio for 20 h. The data shown are the average with SEM values of five repetitions, each including triplicates. Statistical significances were tested in GraphPad Prism ANOVA followed by LSD and **indicates *p *< 0.01 when compared to control T‐cells.

The influence of higher DE as represented by the RS3 WFR considerably impacted the results with greater activation of THP‐1 cells, which were activated in MyD88 dependent and independent ways. Moreover, stronger immune activity by WFR as compared to JD could also be observed in DCs directly exposed to the RS3 in absence and presence of Caco‐SM and on T‐cell skewing.

Moreover, we observed that small changes in degree of polymerization (DP) do not affect immune effects.

### 3.4 High Molecular Weight Drastically Changes Immune Effects of RSs in Relation to Other Traits

The foregoing studies demonstrated that crystallinity in the tested range did not have significant immune effects. However, differences in DE did. Finally, we evaluated the potential of a structurally different RS3, AmyloseV. This pure amylose fraction did not contain amylopectin, unlike the short molecular RSs previously tested, and it therefore differs in crystallinity, DE, and MW. Also, due to the absence of short molecular structures in the AmyloseV sample, DP measurement could not be performed. The comparison of AmyloseV with the most immune active short molecular RS, namely WFR, was used to study the influence of high MW combined with low DE on immune signaling.

**Figure 6:**
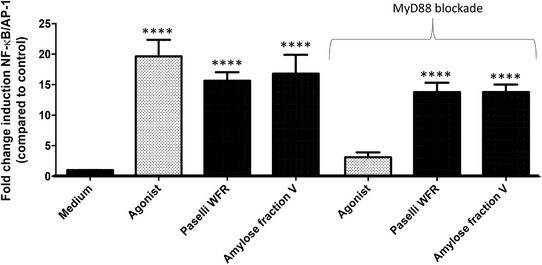
RSs WFR and AmyloseV stimulated monocytic THP‐1 cells in a non‐TLR dependent way. To determine whether immune activation of the two RSs Paselli WFR and Amylose fraction V was TLR‐dependent, we determined activation of THP‐1 cells in fully functional THP‐1 cells (left) and in THP‐1 cells where MyD88 was inhibited by 50 µm Pepinh‐MyD88 (right, as indicated by ‘MyD88 blockade’). Data were normalized so that medium control is 1, and activation levels were expressed as fold change induction of NF‐κB/AP‐1 pathway as compared to medium control ± SEM with *n* = 3 and triplicates. Data were analyzed with GraphPad Prism ANOVA followed by Dunn's test and differences were considered statistically significant when *p < 0.05, **p < 0.01, ***p < 0.001, and ****p < 0.0001.

**Figure 7:**
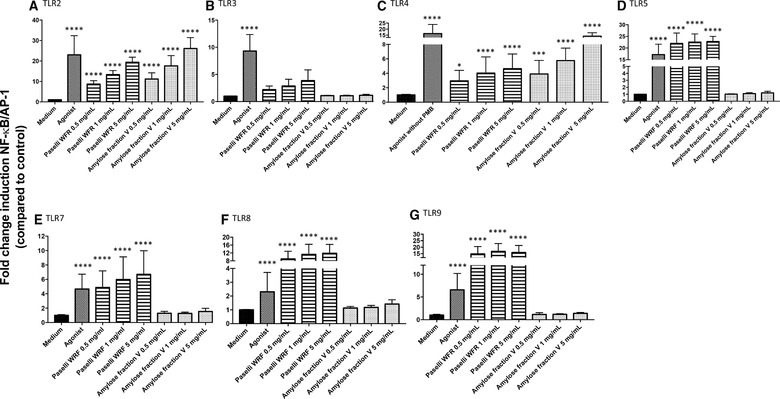
RSs Paselli WFR virtually activated all TLRs and Amylose fraction V only activated TLR2 and 4. WFR had a low crystallinity, high DE, and low MW while AmyloseV had a high crystallinity, low DE, and high MW. TLR signaling was determined by adding these two RSs to various HEK‐cells expressing only one TLR: TLR2 (A), TLR3 (B), TLR4 (C), TLR5 (D), TLR7 (E), TLR8 (F), and TLR9 (G). WFR activated all TLRs and AmyloseV activated TLR2 (A) and TLR4 (C). The activation levels triggered by the RSs were compared to the medium control. Data were normalized so that medium control is 1, and activation levels were expressed as fold change induction of NF‐κB/AP‐1 pathway compared to medium control ± SD with *n* = 5 and triplicates. Data were analyzed with GraphPad Prism Kruskal–Wallis test followed by Dunn's multiple comparison and differences were considered statistically significant when *p < 0.05, **p < 0.01, ***p < 0.001, and ****p < 0.0001.

First, we evaluated the effects of the large molecular AmyloseV on TLR signaling using THP‐1 cells with and without blockade of MyD88. AmyloseV activated THP‐1 cells by 16‐fold (**Figure** 6A), and the activation remained the same despite blockage of MyD88 (Figure 6B), demonstrating that activation is not only TLR dependent. However, by studying effects of AmyloseV on TLR expressing HEK‐cells, we identified that AmyloseV activated TLR2 and 4, in a dose‐dependent manner (**Figure** 7). Activation of TLR2 by AmyloseV was remarkably high with 26‐fold increase (*p *< 0.0001; Figure 7A), while it was only 20‐fold at the same concentration of WFR (*p *< 0.0001). Activation of TLR4 reached 14‐fold with AmyloseV 5 mg mL^–1^ (*p *< 0.0001), that was threefold higher than with WFR (*p *< 0.0001; Figure 7C).

The effects on TLR4 were not caused by possible remnants of LPS in the samples as addition of 100 µg mL^–1^ polymyxin B had no effect on AmyloseV (Figure S3, Supporting Information) induced activation of TLR4.

Exposure of DCs to these two RSs without Caco‐SM showed strong immune effects of AmyloseV. The production of IL‐1β, CCL3/MIP‐1α, CCL‐5/RANTES, IFN‐ γ, and IL‐10 was below detection levels at all occasions. AmyloseV triggered higher average production of CCL‐2 (*p* = 0.0003), IL‐1ra (*p* = 0.0002), IL‐12/23p40 (*p* = 0.021), and TNF‐α (*p* = 0.0006) by DCs as compared to WFR (**Table** 7).

**Table 7:** AmyloseV induces stronger responses of DCs than WFR does. The effects of Paselli WFR and Amylose fraction V on IL‐1Ra. IL‐6, IL‐8, MCP‐1/CCL2, and TNF‐α production was measure by Luminex in spent medium of DCs exposed to RSs directly, DCs exposed to RSs and to Caco‐SM after Caco‐2 were themselves exposed to the RSs, DCs exposed to Caco‐2 spent medium after Caco‐2 were exposed to the RSs, and spent medium from the basolateral compartment of the coculture of DCs with Caco‐2 cells

	Average (pg mL^–1^)	SEM	Average (pg mL^–1^)	SEM	Average (pg mL^–1^)	SEM
**DC‐SM Ingredients**	Medium		Paselli WFR		Amylose fraction V	
CCL‐2/MCP‐1	113.2	7.8	**344.6** ***	44.8	**368.3** ****	72.95
IL‐1ra	1852.0	126.4	2661.0	239.1	**5648.0** ****	1053
IL‐6	5.8	2.1	**73.2** *	24.6	**102.5** **	40.2
IL‐12/23p40	29.0	0.0	132.0	25.2	**247.7** *	43.19
TNF‐α	6.9	1.0	98.1	15.9	**369.8** ****	114.3
IL‐8	420.3	18.0	**3235.0** ****	388.5	**3034.0** ****	340.5
**DC‐SM Caco‐SM with ingredients**	Medium		Paselli WFR		Amylose fraction V	
CCL‐2/MCP‐1	116.8	5.1	**357.0** ****	56.4	**294.7** ***	40.9
IL‐1ra	1733.0	153.3	**3276.0** ****	233.3	**3660.0** ****	378.8
IL‐6	2.9	0.2	**36.6** ****	6.5	**53.4** ****	8.641
IL‐12/23p40			**187.1** ****	10.8	**226.9** ****	3.763
TNF‐α	8.7	0.7	**126.8** ****	22.1	**431.8** ****	45.06
IL‐8	498.4	25.7	**3163.0** ****	381.7	**2520.0** ****	303.4

Caco‐2 cells fully differentiated for 21 days in transwells were stimulated apically with the different compounds at 5 mg mL^–1^ for 20 h. In direct exposure of RSs to the DCs, 5 mg mL^–1^ was used and incubation time was 20 h. The data shown are the average with SD values of five repetitions, each including triplicates. Statistical significances were tested in GraphPad Prsim ANOVA followed by LSD and ****indicates *p *< 0.0001, ***p < 0.001, **p < 0.01, and *p < 0.05 when compared to unstimulated DCs.

When DCs were exposed to the RSs directly in combination with Caco‐SM, AmyloseV induced a strong DC response. AmyloseV led to higher average production of IL‐6 (*p* = 0.024), IL‐12/23p40 (*p *< 0.0001), TNF‐α (*p *< 0.0001), and IL‐8 (*p* = 0.050) by DCs than when they were exposed to WFR (Table 7).

AmyloseV induced T‐cell polarization via modulation of DCs, which was also observed with WFR but not with the other two RSs. Both AmyloseV and WFR increased pleiotropic IL‐6 by tenfold compared to medium controls (**Table** 8).

**Table 8:** RSs WFR and AmyloseV induce increased IL‐6 production by T‐cells. The effects of Paselli WFR and Amylose fraction V on IL‐2, IL‐4, IL‐6, and IL‐10 production were measured by Luminex in spent medium of T‐cells exposed to DCs or coculture spent medium

	Average (pg mL^–1^)	SEM	Average (pg mL^–1^)	SEM	Average (pg mL^–1^)	SEM
**T‐cell DC‐SM Caco‐SM with ingredients**	Medium		Paselli WFR		Amylose fraction V	
IL‐2	66.0	2.9	65.1	2.3	68.17	3.141
IL‐4	2507.0	197.4	2606.0	186.5	2632	240.9
IL‐6	1.7	0.5	**9.1** **	2.5	**11.7** ****	1.259
IL‐10	29.4	5.0	34.0	5.3	28.08	5.26

T‐cells were incubated with spent medium in a 1:10 ratio for 20 h. The data shown are the average with SEM values of five repetitions, each including triplicates. Statistical significances were tested in GraphPad Prism ANOVA followed by LSD and ****indicates *p *< 0.0001 and **p < 0.01 when compared to control T‐cells.

Effects of the lower MW starch WFR were stronger on TLRs while AmyloseV triggered higher response of DCs with and without presence of Caco‐SM. Interestingly, WFR and AmyloseV were strong immune activators, although their molecular structures greatly differed in DE, DP, MW, and crystallinity. Therefore, it seems that the structural traits we identified behind the immune activity of short molecular RS3 (i.e., WFR, JD, and dEtenia in the present study) do not necessarily apply to the category of large molecular RS3 such as the tested AmyloseV. For allowing comparison of the THP‐1 and TLR activating capacity of all four RSs studied here, we have combined the data in Figures S5 and S6, Supporting Information.

## 4 Discussion

In this study, we examined the impact of the structural traits: crystallinity, chain length distribution, and MW on the ability of RS3 to exert direct immune effects. Immune effects of the different RSs were measured via PRR activation, DC cytokines production, with and without presence of IEC medium, and T‐cell polarization. DE seems to be the most important feature influencing immune signaling while crystallinity and MW did not seem to impact immune signaling. Surprisingly, the four tested RSs clearly differed by their ability to activate cells. JD and dEtenia had weak to moderate immune effects. The activation of TLR‐dependent pathways by JD and dEtenia observed in THP‐1 cells was solely due to TLR4 activation, and both these RSs had very limited effects on DC cytokine production. On the other hand, WFR and AmyloseV were strong immune activators. These two RSs activated several PRRs, triggered cytokine production in DCs, and affected T‐cell polarization. Together these results indicate that the structural traits of RSs play an essential role in direct immune stimulation. Within the tested samples, a higher DE combined with lower DP are responsible for these direct effects while crystallinity and MW are of lesser importance.

We show here with the current tested samples that crystallinity and MW are not the main structural features determining capacity of RSs to activate TLRs. This was concluded by first comparing JD and dEtenia, which crystallinities were, respectively, around 7% and 14%. Both these RSs solely activated TLR4. Interestingly, these two RSs originate from the same starch source and underwent only minor changes during production in order to increase the crystallinity of dEtenia as compared to JD. The minor role of crystallinity in TLR activation was further underlined when comparing JD to WFR, as they both have a 7% crystallinity, yet WFR activated all TLRs. Moreover, increasing crystallinity to 92% with AmyloseV confirmed that this parameter is not of great importance, as AmyloseV activated several TLRs too. Also, we found evidence that MW was of minor importance for TLR activation. The strong TLR activator WFR and the weakly activating JD have the same MW, suggesting that MW cannot be the sole determinant for the degree of TLR activation.

In the current set of comparisons, we found that changes in chain length distribution might influence TLR activation. WFR and JD share the same degree of crystallinity and MW but differ in DE, the higher DE being here the stronger immune activator. However, DE is not the only determinant for chain length distribution, which is also closely dependent on DP. This is important as chain length distribution is used to characterize RSs and is known to influence digestibility.^29^ JD had a DP range of 6–20, which is only a minor difference from that of WFR and dEtenia, which ranged from 1 to 22 and 27, respectively. As DP‐values were so similar while WFR had much stronger immune effects compared to the other two small molecular RSs, we feel we can conclude that DP cannot explain the differences in immune stimulating effects. However, the broader and higher DP range was associated with higher DE, which we hold responsible for immune activating activity of RS as WFR with a high DE value activated virtually all TLRs. Although small variations of DP, as tested in our study, do not seem to influence immune activity of small molecular RS3, an association between immune activity and chain length distribution was found.^30^ The broader and higher DP range was linked to higher DE and higher immune activity. WFR with a high DE value activated virtually all TLRs. This was, to the best of our knowledge, never shown before. Interestingly, enhanced IL‐6 production, along with CCL‐2, by DCs was specifically observed, in the present study, with WFR. This illustrates that effects of short molecular RS3 on different cells and/or receptors will be specifically triggered by certain structural traits such as higher chain length distribution and DE in particular.^31^

Our data also suggest that the combination of high MW and high crystallinity could influence DC responses. Induction of anti‐inflammatory IL‐1ra, pro‐inflammatory IL‐12p40, and TNF‐α was specifically observed for AmyloseV, in absence of IECs. This was typical for AmyloseV and not observed with other RSs, not even with the PRR activator WFR. AmyloseV was solely made of amylose and was the largest molecular RS tested in this study. It particularly differed from the other three RSs (WFR, JD and dEtenia) because of its high crystallinity, high MW and low DE. Also, AmyloseV distinguishes itself from the others by the absence of short molecular structures. Therefore, AmyloseV cannot be compared to the other three RSs tested in this study in a step wise fashion, isolating a single structural trait at a time. However, comparing AmyloseV to the other three RSs suggested that conclusions drawn on influence of chain length distribution might only be relevant for short molecular RSs and not for pure amylose fraction, represented here by AmyloseV. DC responses to the exposure to JD and dEtenia, which share the same DE, was unchanged with exception of increased IL‐8 production upon incubation with JD. WFR increased the production of CCL‐2/MCP‐1, IL‐6, and IL‐8. WFR was the most immune active debranched compound, while AmyloseV increased the production of all tested cytokines by DCs. This confirms that, although chain length distribution is likely to influence immune activity of the tested debranched RSs, other traits might be of importance as well.

Furthermore, immune effects of RSs were also assessed on DC cytokine production in presence of IEC medium. Presence of IEC medium after exposure to the RSs strongly influenced DC responses to these RSs. However, none of the investigated structural traits crystallinity, MW, nor chain length distribution seemed to explain the effects observed. dEtenia was unable to stimulate DCs without presence of IEC medium but increased IL‐1ra and IL‐12 production when IEC medium was also added. JD increased IL‐8 production in absence of IEC medium and increased IL‐8 along with IL‐1ra and IL‐12 in presence of IEC medium. This was also observed with WFR. Conditioning of DC responses by IEC medium has been reported before^14^, ^15^, ^16^, ^31^ and was shown to be essential to study RS effects on DC responses.^4^ In this study, RS was reported to decrease IL‐12 production in presence of IECs. This, the current, but also another study showed that IEC medium conditioned DCs toward a noninflammatory state,^15^, ^16^ therefore, confirming the importance of taking crosstalk into account. Moreover, this did not apply to AmyloseV, which was highly immune active both in presence and absence of IEC medium.

The high immune activity of WFR was also observed on PRR activation and T‐cell polarization while the other two debranched RSs had no effects confirming previous findings about its direct effects on immune receptors and cells. To a lesser degree, this was also observed for the fractionated RS AmyloseV, which activated both TLR2 and 4 while WFR activated virtually all TLRs. This could only partly be explained by LPS contamination as addition of polymyxin B, i.e., an LPS inactivator, had limited effects on TLR activation. Interestingly, WFR and AmyloseV contained, although in different amount, some amylose that might correlate with the activation of TLR2 and 4. A previous study described activation of TLR2 by two different RSs including a RS3,^4^ and, as also observed in our study for WFR and AmyloseV, other PRRs than just TLRs. Interestingly, it seems that activation of more than two TLRs by a single RS molecule, as observed for WFR, is unique and dependent on structural traits such as chain length distribution.

Effects observed on T‐cells were similar for WFR and AmyloseV, although AmyloseV displayed unique effects on DCs by increasing the production of virtually all cytokines tested. AmyloseV and WFR both activated TLR2, however AmyloseV, in absence of IEC medium, is the only RS that activated IL‐1ra, IL‐12, and TNF‐α. We have no explanation for this unique effect of AmyloseV, but release of cytokines, by DCs, similar to those present in IEC medium might be a possible mechanism. WFR and AmyloseV are the only two RSs that induced T‐cell polarization, as observed by increased IL‐6 production. It is possible that this is due to the TLR2 activating capacity of these RSs. TLR2 activation is involved in induction of Treg^4^, ^32^ and shaping a tolerant microenvironment at mucosal sites,^33^ which corroborates our finding that production of pleiotropic IL‐6 by T‐cells is increased. Importance of chain‐length distribution on TLR2 activation was previously reported for other dietary fibers.^30^ However, it cannot relate to AmyloseV effects. It seems that WFR and AmyloseV both display unique and great effects on different levels of immune responses and that different structural traits are responsible for these effects.

In conclusion, our results show that all tested RSs WFR, JD, dEtenia, and AmyloseV can interact with TLRs in a dose‐dependent manner and that chain length distribution might be determinant for differences in effects. Most importantly, we show that immune activity of RS3 are category dependent. Short molecular starches mostly composed of amylopectin differ from larger molecular starches solely made of amylose. AmyloseV is the large molecular starch we had available for our study. This large molecular AmyloseV strongly supported immunity, and its effects were most specific on stimulation of cytokine production by DCs even in absence of Caco‐SM, in the contrary of short molecular RSs. Within the category of short molecular RSs, a higher chain length distribution, which was characteristic for WFR, with a stronger difference on DE than DP, was associated with stronger and broader PRR activation, DC cytokines, and T‐cell IL‐6 production as compared to the other two short molecular RSs. Although no characteristic structure could be related to the effects observed on T‐cells, TLR2 activation is involved, as both WFR and AmyloseV induced a similarly strong increase of IL‐6 production by T‐cells and activated TLR2. Further investigation is therefore crucial to determine the mechanisms of action of RSs, however we have shown for the first time, to the best of our knowledge, that responsible traits for immune activity among short molecular RS3 are chain length distribution and DE in particular. These traits determined PRR activation. Amylose content that is presented in WFR in very low amounts and is the main molecule present in AmyloseV enhanced DC and T‐cell responses. Although crystallinity and MW did not impact PRR activation in debranched small molecular RSs, a combination of high crystallinity and MW might be key in direct DC stimulation as observed with AmyloseV. This knowledge may be used for tailoring RS formulations for immune stimulation such as during pathogenic infections^34^ or for support of immunity in elderly.^35^

## Conflict of Interest

The authors declare no conflict of interest.

## Supporting information

Supplementary
