# Supplementary material for: Higher Chain Length Distribution in Debranched Type‐3 Resistant Starches (RS3) Increases TLR Signaling and Supports Dendritic Cell Cytokine Production
Source: Mol Nutr Food Res. 2018 Nov 23;63(2):1801007. doi: 10.1002/mnfr.201801007 (PMC6767581; doi:10.1002/mnfr.201801007)
Supplement: Supplementary file 1 — Supplementary [file MNFR-63-na-s001.docx]

**Supportive Figures**


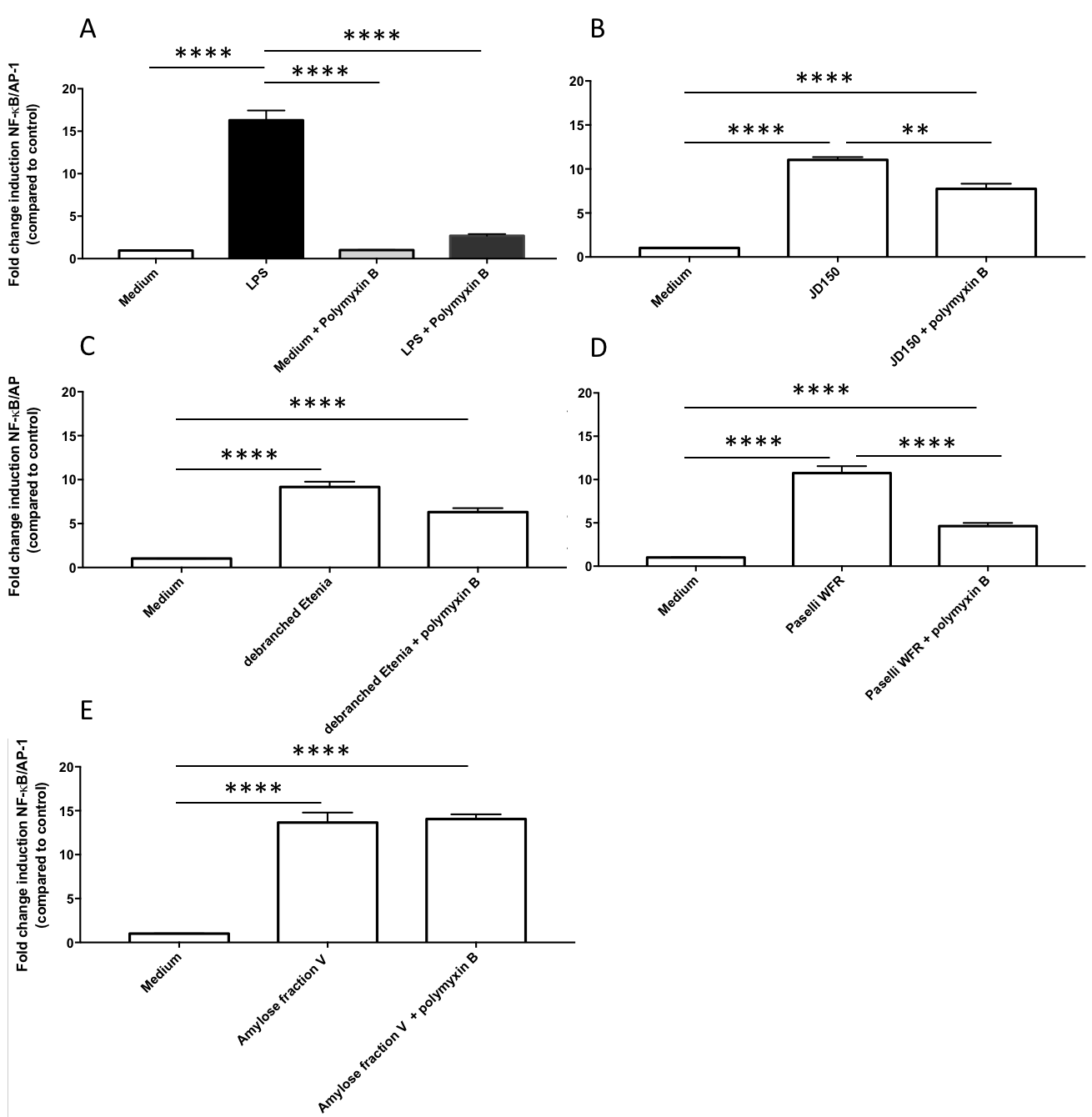


**Figure S1. TLR4 activation by JD and dEtenia is not an artefact due to LPS contamination.** The effect of polymyxin B on TLR4 activation by the agonist LPS (A) and the effect of the RSs on TLR4 activation in absence and presence of polymyxin B shows that possible endotoxin contamination is efficiently counteracted by coincubation with 100 µg/mL polymyxin B for JD150 (B) and debranched Etenia (C). Statistical significance compared to agonist control were calculated in Graphpad Prism using Kruskal Wallis followed by Dunn’s test with ** *p*<0.01 and **** *p*<0.0001.


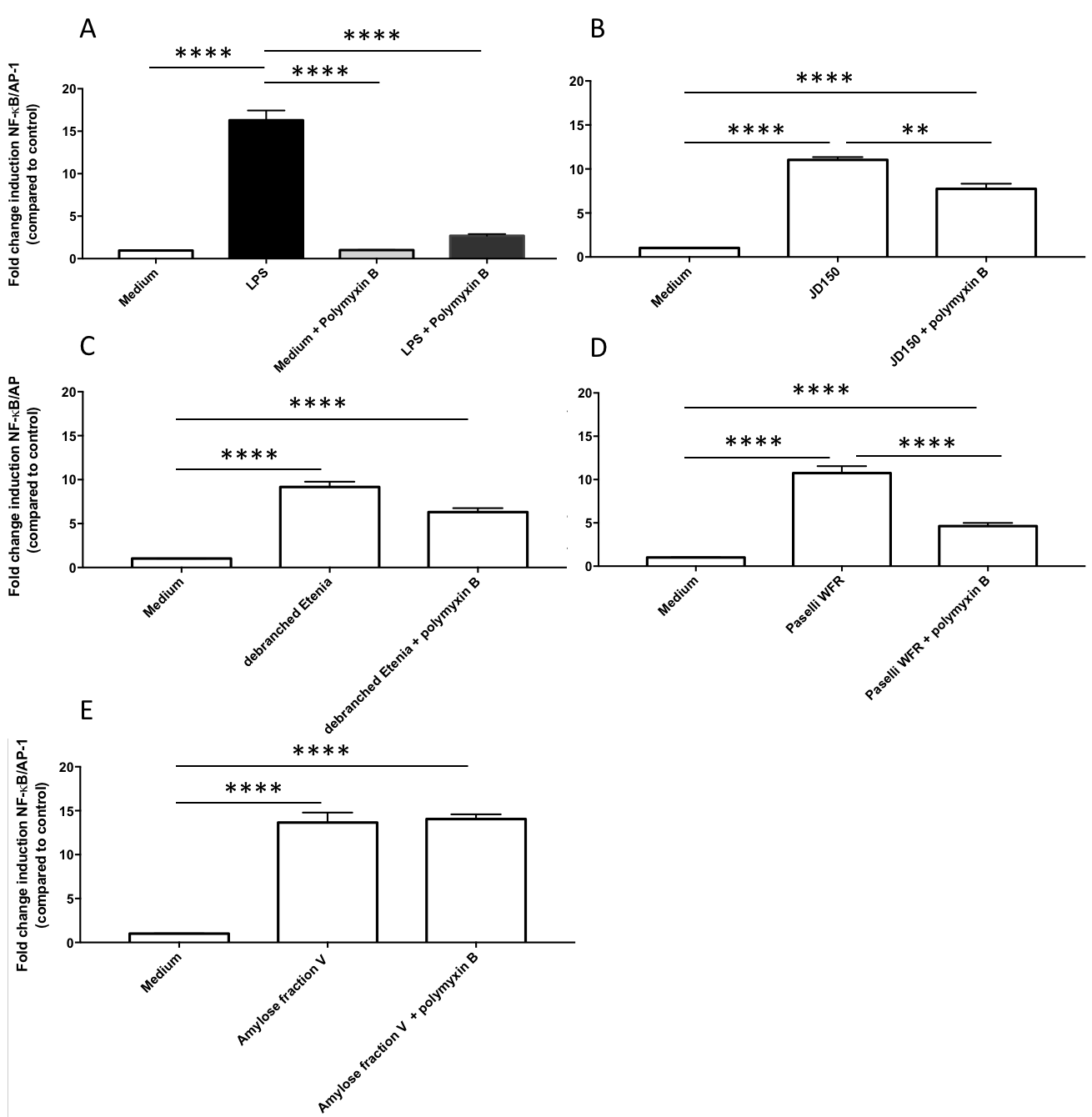


**Figure S2. TLR4 activation by WFR is not an artefact due to LPS contamination.** The effect of Paselli WFR on TLR4 activation in absence and presence of with 100 µg/mL polymyxin B shows that Paselli WFR does activate TLR4 independently of possible endotoxin contamination. Statistical significance compared to agonist control were calculated in Graphpad Prism using Kruskal Wallis followed by Dunn’s test with **** *p*<0.0001.

**
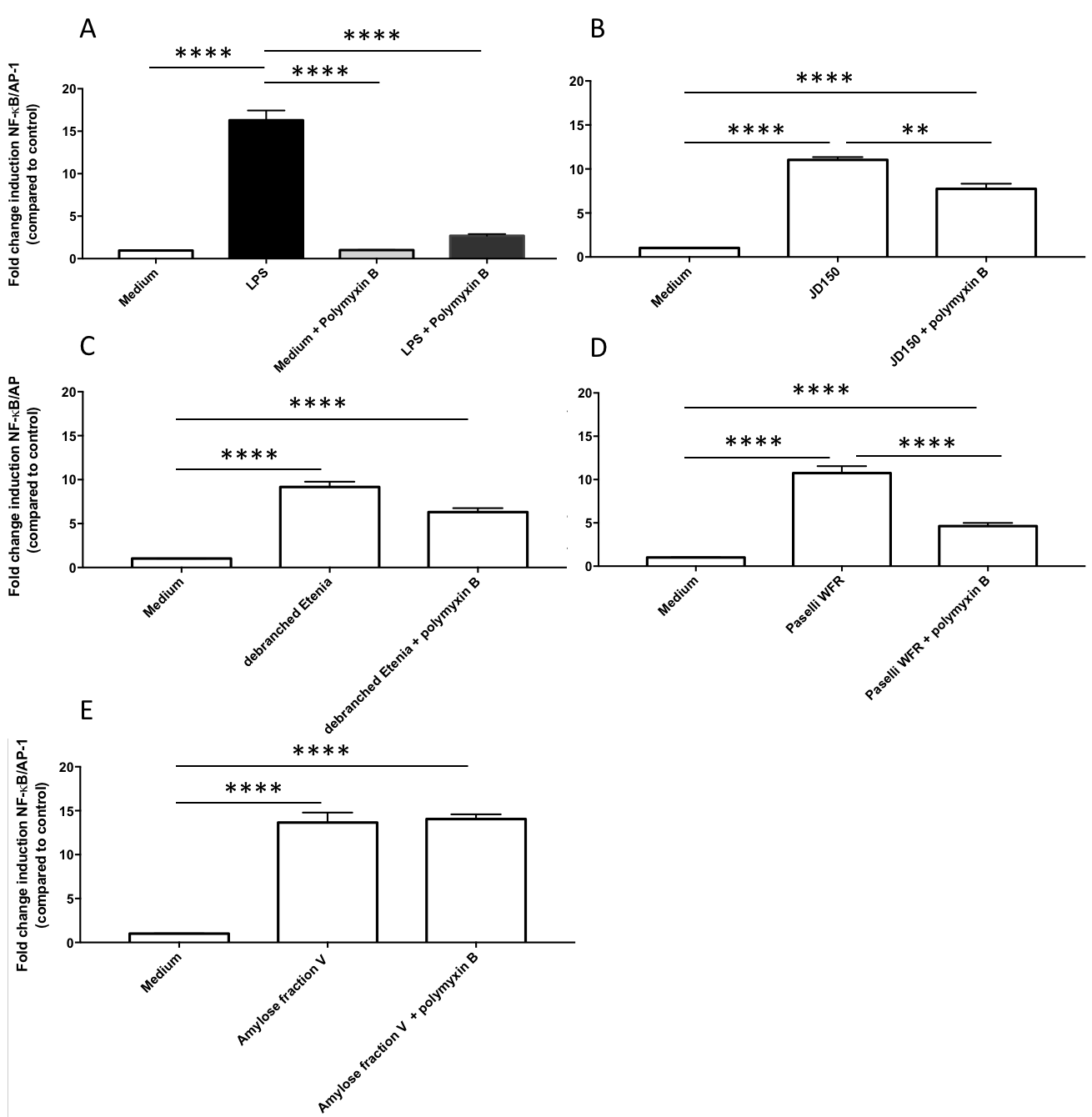
**

**Figure S3. TLR4 activation by AmyloseV is not an artefact due to LPS contamination.** The effect of Amylose fraction V on TLR4 activation in absence and presence of with 100 µg/mL polymyxin B shows that Amylose fraction V does activate TLR4 independently of possible endotoxin contamination. Statistical significance compared to agonist control were calculated in Graphpad Prism using Kruskal Wallis followed by Dunn’s test with **** *p*<0.0001.


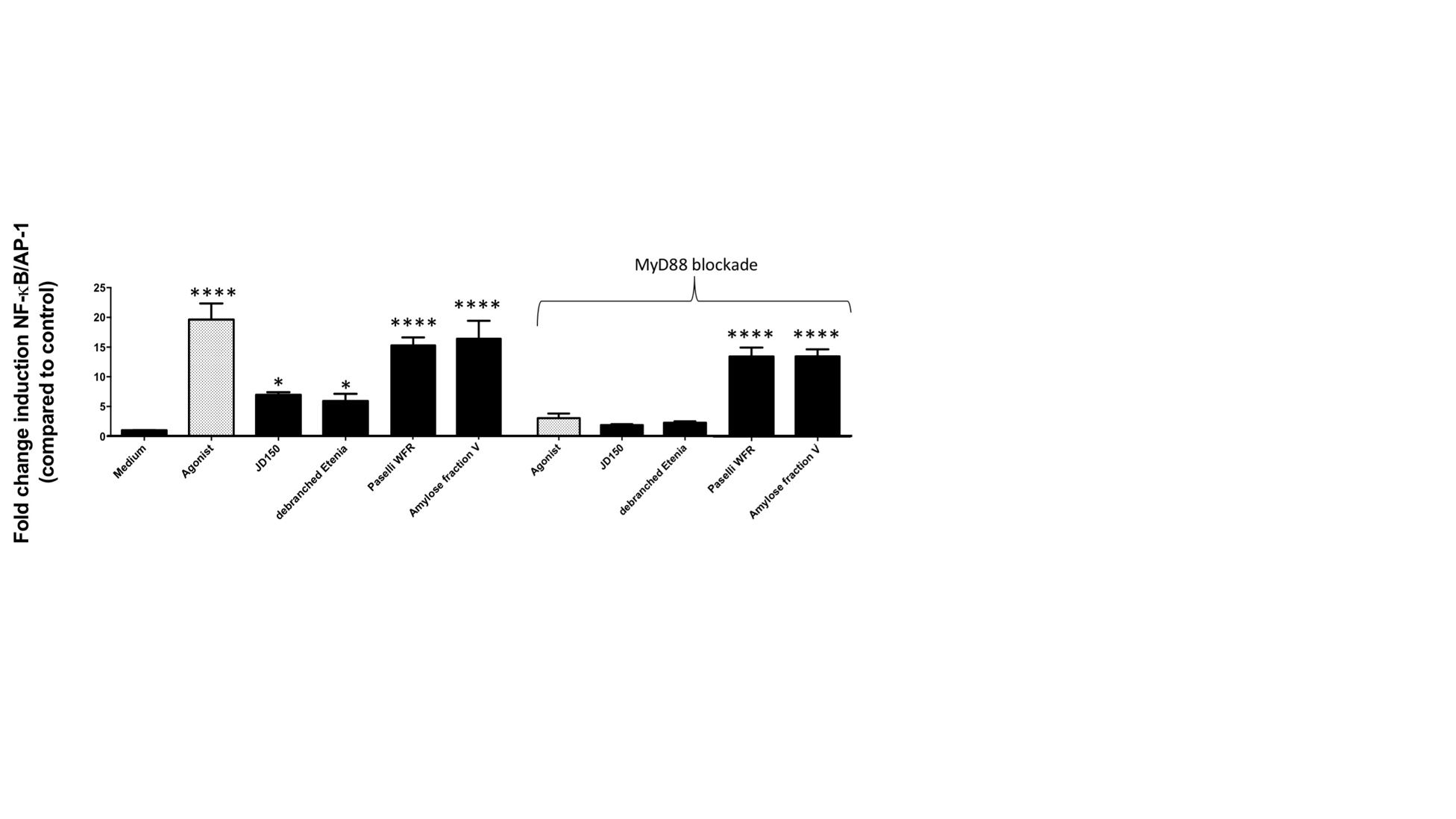


**Figure S4. PRR activation by the four tested starches.** To determine whether immune activation of the three short molecular RSs JD, dEtenia, WFR and the large molecular RS AmyloseV was TLR dependent, we determined activation of THP-1 cells in fully functional THP-1 cells (left). We also used THP-1 cells where MyD88 was inhibited by 50 µM Pepinh-MyD88 (right, as indicated by ‘MyD88 blockade’). Data were normalized so that medium control is 1, and activation levels were expressed as fold change induction of NF-κB/AP-1 pathway as compared to medium control ± SEM with n=3 and triplicates. Data were analyzed using GraphPad Prism ANOVA test followed by LSD test, and differences were considered statistically significant when *p*<0.05 *, *p*<0.01 **, *p*<0.001 *** and *p*<0.0001 ****.


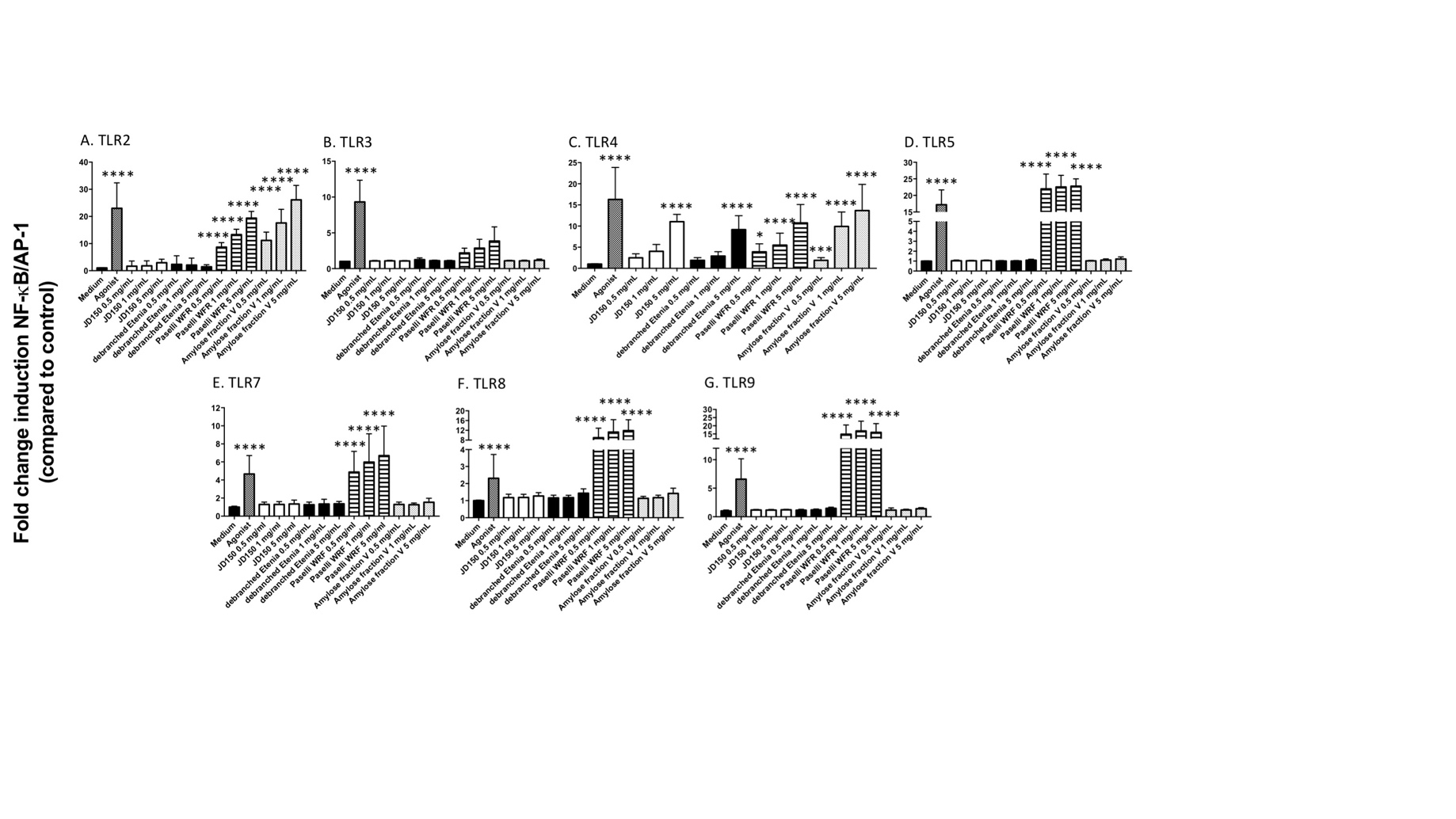


**Figure S5. TLRs activation by the four tested starches.** TLR signaling was determined by adding the RSs JD, WFR, dEtenia and AmyloseV to various HEK-cells expressing only one TLR. WFR and Amylose activated TLR2 (A), none activated TLR3 (B), all activated TLR4 (C), WFR was the only one to activate TLR5 (D), TLR7 (E), TLR8 (F) and TLR9 (G). The activation levels triggered by the RSs were compared to the medium control. Data were normalized so that medium control is 1, and activation levels were expressed as fold change induction of NF-κB/AP-1 pathway as compared to medium control ± SD with n=5 and triplicates. Data were analyzed with GraphPad Prism Kruskal-Wallis test followed by Dunn’s multiple comparison and differences were considered statistically significant when *p*<0.05 *, *p*<0.01 **, *p*<0.001 *** and *p*<0.0001 ****.
